# Isolation and characterization of exosomes for cancer research

**DOI:** 10.1186/s13045-020-00987-y

**Published:** 2020-11-10

**Authors:** Le Zhu, Hao-Ting Sun, Shun Wang, Sheng-Lin Huang, Yan Zheng, Chao-Qun Wang, Bei-Yuan Hu, Wei Qin, Tian-Tian Zou, Yan Fu, Xiao-Tian Shen, Wen-Wei Zhu, Yan Geng, Lu Lu, Hu-liang Jia, Lun-Xiu Qin, Qiong-Zhu Dong

**Affiliations:** 1 Department of General Surgery, Huashan Hospital, Cancer Metastasis Institute, Fudan University, 12 Urumqi Road (M), Shanghai, 200040 China; 2 Institutes of Biomedical Sciences, Fudan University, 131 Dong An Road, Shanghai, 200032 China; 3 Fudan University Shanghai Cancer Center, Shanghai Medical College, Fudan University, Shanghai, 200032 China

**Keywords:** Exosomes, Cancer, Isolation, Characterization, Biomarker, Therapy

## Abstract

Exosomes are a subset of extracellular vesicles that carry specific combinations of proteins, nucleic acids, metabolites, and lipids. Mounting evidence suggests that exosomes participate in intercellular communication and act as important molecular vehicles in the regulation of numerous physiological and pathological processes, including cancer development. Exosomes are released by various cell types under both normal and pathological conditions, and they can be found in multiple bodily fluids. Moreover, exosomes carrying a wide variety of important macromolecules provide a window into altered cellular or tissue states. Their presence in biological fluids renders them an attractive, minimally invasive approach for liquid biopsies with potential biomarkers for cancer diagnosis, prediction, and surveillance. Due to their biocompatibility and low immunogenicity and cytotoxicity, exosomes have potential clinical applications in the development of innovative therapeutic approaches. Here, we summarize recent advances in various technologies for exosome isolation for cancer research. We outline the functions of exosomes in regulating tumor metastasis, drug resistance, and immune modulation in the context of cancer development. Finally, we discuss prospects and challenges for the clinical development of exosome-based liquid biopsies and therapeutics.

## Background

Exosomes, which are secreted by multiple cell types, are a subtype of extracellular vesicles (EVs) that range in size from approximately 40 to 160 nm in diameter [1]. Tumor cells have been found to robustly produce and secrete exosomes [2, 3]. Exosomes have been found in multiple bodily fluids, including blood, lymph, urine, cerebrospinal fluid, bile, saliva, and milk (among others). Exosomes were first discovered by Pan and Johnstone in the 1980s as endocytic microvesicles released by maturing reticulocytes [4, 5]. Exosomes have traditionally been considered cellular “trash bags”, a simple means for disposing of unnecessary cellular waste products. It was not until the mid-1990s that exosomes were gradually demonstrated to play vital roles in intercellular communication in normal physiological processes and in the pathogenesis of disease, including cancer [6, 7]. Exosomes carry membranous and cytoplasmic substances derived from their parental cells, such as proteins, messenger RNAs (mRNAs), microRNAs (miRNAs), long non-coding RNAs (lncRNAs), lipids, metabolites, and even DNA fragments [8, 9]. Surface receptors on exosomes allow them to be targeted to and captured by recipient cells. Increasing evidence has confirmed that exosomes can be transferred from host to recipient cells, leading to the exchange of genetic information and reprogramming of cellular functions [7]. They can interact with receptors on local or distant target cells and modulate signaling pathways. Exosomes can also modify the physiological state of target cells by releasing their specific contents after endocytosis via phagocytosis or via direct fusion with the cell membrane [10]. Therefore, exosomes have been recognized as intercellular interaction mediators that can regulate various biological functions [11, 12]. In the context of cancer, exosomes are involved in a wide range of processes that underlie cancer progression, e.g., regulation of tumor metastasis, development of drug resistance, and immune modulation [13–15].

In addition to exosomes, cells produce other types of EVs, including microvesicles (MVs) and apoptotic bodies, which are differentiated based on their biogenesis, size, physical properties, content, and function [16]. As a prerequisite to fundamental research and biomarker discovery using exosomes, they must be isolated from non-exosomal components in sufficient quantity and purity based on size, biochemical properties, and surface markers [17]. A number of techniques have been developed for exosome isolation, including ultracentrifugation (UC), filtration, size-exclusion chromatography (SEC), immunoaffinity capture [18, 19], and microchip-based techniques [20], all of which have distinct isolation principles and unique sets of advantages and disadvantages [21]. Moreover, many kinds of commercial kits are available for exosome isolation [21]. However, due to the uncertain quality of exosome preparations, the quality and efficiency of exosome isolation still requires further improvement and assessment [22, 23]. Currently, effective and accurate separation of highly pure exosomes remains a significant challenge due to their nanoscale size and substantial heterogeneity [24].

The ability to frequently monitor cancer progression and to assess treatment efficacy early could inform clinical decision-making and design of personalized cancer treatments. Exosomes are highly heterogeneous [25] and contain molecular signatures reminiscent of their cell of origin. Exosomes isolated from patient biofluids have been shown to contain cancer-specific cargo reflecting altered cellular or tissue states [26, 27]. These findings have raised the idea that the analysis of the molecular content of exosomes could provide unique opportunities in the context of liquid biopsies for gaining information about the presence, molecular profile, and behavior of cancer. Therefore, exosomes can be used as biomarkers in liquid biopsies for real-time monitoring of tumor burden and treatment efficacy [28].

The field of exosome-based cancer therapeutics was first established by Thery and colleagues 20 years ago in two publications highlighting the potential of exosomes as therapeutic cell-free vaccines in anticancer vaccine development [29, 30]. Since then, the potential to use exosomes as therapeutic agents has become an exciting and rapidly evolving research field [31]. It is well known that exosomes consist of a lipid bilayer membrane that naturally protects them from clearance or degradation in the circulation. Remarkably, exosome release and uptake occur naturally, and because they possess intrinsic cell-like properties they can overcome natural barriers, such as the blood–brain barrier (BBB). Hence, exosomes can also potentially be used as drug delivery vesicles for treating disease, including cancer [32]. Furthermore, exosome engineering, i.e., modification of exosomes to carry a defined range of contents, may provide opportunities to enhance or broaden their therapeutic capability in clinical settings [33]. Considerable challenges remain to be overcome in the development of novel cancer therapeutic strategies; therefore, exosome-based cancer therapeutics are heralded as an attractive approach in the precision oncology paradigm.

In this review, we discuss the biogenesis, release, isolation, characterization, and biological functions of exosomes as well as their clinical application and challenges related to technical and biological issues and clinical translation. It is hoped that new strategies and exosome-based approaches might help researchers devise novel therapeutic treatments to limit cancer progression.

## Exosome isolation methodologies

Current characterization of the biological activities of exosomes has largely relied on diverse EV isolation methods. Therefore, it is imperative to be able to quickly and reliably separate exosomes from a wide range of cell debris and other EVs. Based on the size and affinity of exosomes, different isolation strategies can be used to isolate them from biofluids or cell culture supernatant. Unlike techniques used for isolating nucleic acids and proteins, techniques for exosome isolation have only been developed in the past few decades. The size similarity between exosomes and other EVs, which include ectosomes and MVs, has deeply impeded the development of isolation processes. In recent decades, an increasing number of techniques for exosome isolation have been explored [21]. These techniques can be broadly classified based on their key mechanism: UC, density gradient (DG) centrifugation, infiltration techniques, immunoaffinity, capture-based techniques, exosome precipitation, and use of acoustic nanofilters (Table 1).

**Table 1:** Comparison of different exosome separation technologies

Separation technology		Advantages	Disadvantages	Refs
Centrifugation techniques	Ultracentrifugation	Most commonly used and well developed	Low purity	[46, 60]
	Density gradient centrifugation	High practicability	Time consuming	[46, 61]
Size-based techniques	Ultrafiltration	Size uniformity of yield	Low yield and potential for pore blockage	[53, 62]
	Size exclusion chromatography	Economical and non-destructive	Complicated	[63, 64]
Capture-based techniques	Magnetic beads and immunoaffinity	High purity Specific separation	Separate exosomes with targeted proteins only	[45, 54, 65]
Polymer-based techniques	Commercial kits	Fast procedures Convenient operation	Unstable quality of kits Massive expense	[57, 66]
Microfluidics-based techniques	Size-based microfluidics Immunoaffinity-based microfluidic separation Dynamic microfluidics	Fast separation Continuous process Higher purity	Complicated equipment Difficult to operate	[35, 55, 56, 67]

### Ultracentrifugation techniques

UC is the most used isolation method, and it plays a crucial role in the process of exosome isolation. DG centrifugation, which is a derivative of UC, is considered the “gold standard” for exosome isolation [34]. Upon high-speed centrifugation with successive centrifugation parameters, dead cells, cellular debris, and apoptotic bodies are efficiently removed, and a broad range of exosomes can be separated based on their pelleting properties. Traditional UC is widely used because of its usefulness with biofluids, including serum, urine, cerebrospinal fluid, breast milk, aqueous humor, and amniotic fluid [35]. During the development of new exosome isolation methods, UC became the most frequently used method for exosome isolation from cell culture supernatant and biological fluids before 2015 [36]. However, the yield and purity of exosomes isolated via UC greatly depend on many factors, including rotor type, centrifugation time, and sample viscosity [37, 38]. Correspondingly, these parameters should be considered when using and optimizing UC protocols for particular types of samples. It is known that DG centrifugation enables the separation of subcellular components and increases the efficiency of particle separation according to their buoyant density [39]. DG centrifugation is used to separate exosomes depending on differences in size and density between the exosomes and other components, which usually require different centrifugation forces and times for pelleting. DG centrifugation has been extensively used with a variety of samples, including plasma, cell culture supernatant, serum, saliva, urine, and breast milk. For example, DG centrifugation has been used to extract EVs, including exosomes, from salivary fluid, a mixture of gland secretions, gingival crevicular fluids, cell debris, and microorganisms [34]. Although this method is easy to perform and yields exosomes with higher purity, the process is time consuming and highly instrument dependent. Recent studies reported that repeated UC leads to low-yield exosomes and adverse effects on exosome quality, which are incompatible with clinical applications. Furthermore, it has been reported that this method can yield potentially damaged exosomes, most likely due to the high shear forces experienced by the exosomes during high-speed centrifugation [40, 41].

### Size-based techniques

There are three main types of size-based techniques, i.e., ultrafiltration, sequential filtration, and SEC. Ultrafiltration, characterized by a 10–100 kDa molecular weight cutoff (MWCO), is commonly used as a first step to concentrate exosomes from large volumes of original material into small-volume samples that can be used in subsequent purification procedures [42]. The process of sequential filtration is usually divided into three steps. In the first step, cells and cellular debris are filtered; next, free proteins are depleted, and the samples are concentrated; finally, exosomes are sorted using filters with specific, defined pore sizes [43]. Compared with centrifugal and filtration methods, SEC has multiple advantages, including reproducibility, cost-effectiveness, and its non-destructive outcomes. Importantly, this methodology is also compatible with exosome extraction from serum and plasma. An advanced ultrafiltration, sequential centrifugal ultrafiltration (SCUF) approach has also been used to obtain highly pure exosomes and to sieve out MVs from a human colon cancer cell line [44]. Recently, a study revealed that ultrafiltration is a better alternative to UC as it showed the highest recovery of particles of less than 100 nm, which included exosomes. NanoSight and transmission electron microscopy (TEM) showed that the size distributions of exosomes isolated via UC or SEC were similar. Compared with the classical UC protocol, ultrafiltration techniques provide a higher particle yield, thereby increasing exosome yield and isolation efficiency with a shorter processing time. While these size-based techniques have been widely used in many fields, they still require a relatively long running time, limiting their usefulness in treatment and research.

### Capture-based techniques

Capture-based techniques, which are closely related to immunoaffinity, are often used to produce high-purity exosomes [45]. It is very important to note that magnetic beads, a novel tool that can be modified to bind to target proteins on membrane surfaces, play a central role in capture-based techniques. The surfaces of exosomes contain a variety of membrane proteins, such as CD9, CD63, ALIX, and Ep-CAM, which can be enriched using antibody-coated magnetic beads [46]. Depending on the specific immune interaction between the antibody and antigen, the process of collecting immobilized specific exosomes can be successfully achieved via washing in a stationary phase. This technique meets the rigorous demands of separating exosomes that contain specific target membrane proteins. The conclusion that capture-based techniques involving the Ep-CAM biomarker represent the best approach for separating exosomes in comparison with other methods has been widely accepted due to comprehensive analyses of the efficiency of recycling exosomes [47]. Recently, a study revealed that an approach for isolating EVs from urine using the Vn96-peptide, which specifically binds to EVs containing a heat shock protein, is much faster than traditional methods in prostate cancer, such as UC [48]. While the mechanism of this heat shock-based isolation methodology is not clear, it is unquestionably conducive to the development of advanced methodologies useful not only for prostate cancer but also for other malignant tumors. The IAC-Exo methodology, which involves specific immunoaffinity and magnetic bead capture mechanisms, is the most efficient technique for exosome enrichment compared with DG centrifugation and UC. Since the amount of exosomes captured via immunoaffinity is at least twofold higher than the amounts recovered using the other two methods, IAC-Exo has been proposed for wide use in fields concerned with exosome-based treatment and research [46]. The exoRNeasy Serum/Plasma Kit (Qiagen, Hilden Germany), a membrane-based affinity binding technique, has been widely used in purification of total exosome-derived RNA from serum/plasma. Therefore, this commercial kit undoubtedly represents a methodology leveraging capture-based techniques [49, 50].

As it is based on immunoaffinity isolation, this technology makes it possible to separate distinct exosome subpopulations produced by specific cell types to study differences in the functional effects of exosome subpopulations. Furthermore, this technology allows visualization of individual exosomes and detection of protein markers on single exosomes. Unfortunately, magnetic bead-based separation strategies are not suitable for large-scale exosome separation. In addition, high cost and low yield limit their further development and use.

### Precipitation techniques

Unlike the above isolation methods, the mechanism of precipitation techniques mainly depends on the use of polymers to precipitate exosomes, which are then prepared for further purification. Polyethylene glycol (PEG), the most common polymer used in exosome isolation, robustly promotes enrichment and increases exosome yield [51]. Before its use with exosomes, this method was reported to be feasible for isolating various biomolecules as well as virus from bodily fluids [52]. In this method, samples are co-incubated with PEG solution at 4 ℃ overnight. After this incubation, a series of separation steps, such as filtration and centrifugation, can be used to further process the exosome-containing precipitate. With the growing demand for increased efficacy and efficiency in exosome isolation processes, more and more biotech companies are paying great attention to developing commercial products for exosome isolation, including ExoQuick (System Biosciences, United States), Total Exosome Isolation Reagent (Invitrogen, United States), ExoPrep (HansaBioMed, Estonia), Exosome Purification Kit (Norgen Biotek, Canada), and miRCURY Exosome Isolation Kit (Exiqon, Denmark) [35]. However, commercial exosome isolation kits vary in efficiency and exosome quality. Studies have demonstrated that compared with two other polymer-based kits (ExoQuick™ or OptiPrep™); the Exo-spin™ kit is the best commercial approach for exosome extraction due to its higher quality and purity of yield [53]. Precipitation-based methods for exosome isolation are the most attractive for clinical research due to their simplicity and speed, lack of exosome damage, and the low demand for additional equipment for isolation. However, it has been reported that these methods suffer from co-isolation of various contaminants from the sample, including non-exosomal proteins (e.g., albumin) and other particles [54]. Therefore, heavy contamination with plasma proteins limits the utility of precipitation techniques for proteomic analysis of exosomes from human plasma. In addition, exosomes isolated via precipitation methods might contain biopolymers that can complicate further sample analysis, including mass spectrometry, proteomic analysis, and RNA assays. However, the addition of an efficient pre-filtration step through a 0.22-µm filter or a post-precipitation purification step, including subsequent centrifugation, filtration, or gel filtration, can limit contamination with non-exosomal impurities from the samples [53]. Modern precipitation methods are attractive for clinical applications because they require very little starting material when working with human biofluids and are compatible with high-throughput options.

### Microfluidics-based techniques

Microfluidics systems are an ideal tool for separating exosomes from other nanometer-sized particles since they support cost-efficient, high-speed, and precise isolation processes [55]. Microfluidics-based techniques are known for their unique properties, including low cost and low time demand. In addition to these advantages, these techniques also solve a crucial problem: they avoid the non-continuous separation processes involved in other common methods. Currently, widely used microfluidics tools are fully integrated with size-based separation, immunoaffinity-based separation, and dynamic separation. In recent years, an emerging exosome isolation technique, the ExoTIC device, was introduced. The popularity of the ExoTIC device gradually increased due to its undisputed advantages, including high yield, purity, and efficiency. When compared with PEG precipitation (including the ExoQuick™ method) and UC, the ExoTIC device is more amenable for extracting exosomes from serum or other bodily fluids [56]. Despite its numerous advantages, including high purity, controllability, isolation specificity, and high efficiency, there remain some problems, including the requirement for complicated devices for isolation and limitations based on the need for high immunoaffinity [57]. In addition to their development for exosome isolation, microfluidic platforms have also been extensively developed for DNA, protein, and virus separation. While there are many foreseeable challenges, microfluidics-based techniques will be explored for broad use in procedures focused on the isolation of various bioactive molecules, including exosomes [58, 59].

Above all, an ideal method for exosome isolation should be relatively simple, fast, efficient, inexpensive, and scalable. It should also not damage the exosomes or require additional equipment. In fact, various methods have specific advantages and disadvantages in terms of efficiency, reproducibility, and impact on functional outcomes. Further optimization of isolation protocols and the use of combinations of isolation techniques may help overcome these disadvantages and accelerate exosome research for both basic and clinical applications.

## Characteristics of exosomes in cancer

### Exosome biogenesis

Exosome biogenesis was first observed during sheep reticulocyte maturation as exosomes were secreted into the extracellular environment [4, 68, 69]. Exosome biogenesis involves double invagination of the plasma membrane and the formation of multivesicular bodies (MVBs) that contain intraluminal vesicles (ILVs). ILVs are eventually secreted as exosomes via fusion of MVBs with the plasma membrane and exocytosis. The first invagination of the plasma membrane involves cell-surface proteins and soluble proteins and leads to de novo formation of early-sorting endosomes (ESEs). With help from the trans-Golgi network and the endoplasmic reticulum, ESEs mature into late-sorting endosomes (LSEs) and ultimately generate MVBs [7, 70–72]. The second invagination of the endosomal-delineating membrane leads to MVB formation. This process yields MVBs that contain several ILVs, which ultimately become exosomes. Next, MVBs can either fuse with lysosomes or autophagosomes to be recycled, or they fuse with the plasma membrane to secrete the existing ILVs as exosomes [71, 73, 74]. Evidence has proven that the endosomal sorting complex required for transport (ESCRT) participates in ILV formation. Four separate ESCRT subunits (0 through III) work cooperatively to promote MVB formation, vesicle budding, and protein cargo sorting [75–77]. It has been demonstrated that the ESCRT-0 subunit of the complex recruits proteins for internalization, including ubiquitinated proteins and clathrin; that ESCRT-I and ESCRT-II initiate the beginning of the budding process and facilitate enzymatic de-ubiquitination of cargo proteins; and that ESCRT-III is involved in the final stage of membrane invagination and separation [78, 79]. In addition, the typical exosomal protein Alix has been demonstrated to promote endosomal membrane budding and abscission as well as exosomal cargo selection via an interaction with syndecan [80]. Depletion of the ESCRT complex has been shown to reduce the number of MVBs without completely eliminating them, demonstrating the existence of ESCRT-independent mechanisms. Studies have shown that both ceramide-rich lipid domains and tetraspanin CD63 on the extracellular side of the membrane are essential for ILV formation [74, 81, 82]. The efficiency of the transformation of sphingomyelin into ceramide can also influence exosome biogenesis [83, 84]. Recently, emerging research has demonstrated that LC3 mediates exosome release via an LC3-dependent process of EV loading and secretion (LDELS). In addition, LDELS can also regulate the content of exosome-derived RNA in samples from biofluids [85].

### Exosome composition

The composition of exosomes is to some extent cell-type dependent and can also be affected by different cellular states. In 2007, exosomes were first reported to contain both mRNA and miRNA [86]. Since then, many groups have confirmed that exosomes also carry a multitude of non-coding RNA (ncRNA) species, including miRNA, circRNA, and lncRNA [87, 88]. Some studies have shown enrichment of specific RNAs in exosomes that differ from the RNA composition of the donor cells, demonstrating the existence of an RNA sorting process during exosome formation [89, 90]. It has been proven that mRNA molecules transported by exosomes can be translated into protein, demonstrating the potential for horizontal transfer of material between cells [86]. In addition, other types of RNAs, including ncRNAs, are also functional in exosomes and can impact the transcriptome of recipient cells [91–93]. RNA binding proteins (RBPs) encapsulated in exosomes maintain the normal structure and function of RNAs and prevent their hydrolytic degradation [94]. Via protection by exosomes, bioactive RNAs can exert effects via cell-to-cell communication [95]. Abundant studies have described the RNA component of exosomes, but relatively less is known about the composition of genomic DNA (gDNA). During the past few years, several studies have confirmed the presence of gDNA fragments and mitochondrial DNA (mtDNA) in exosomes [96–98]. The gDNA content varies significantly between tumor cell-derived exosomes and exosomes isolated from blood and ascites (Aex). Under treatment with genotoxic drugs, nuclear components, including micronuclei (MN), can be encapsulated by exosomes [99]. However, studies have shown that dsDNA and histones cannot be transported by exosomes [94]. For now, the physiological significance of DNA in exosomes remains unclear, and further investigation is required [94]. A variety of proteins have been observed in exosomes, including cytoskeletal proteins, tetraspanins (CD9, CD63, CD81 and CD82), ESCRT-associated components (Alix and TSG101), heat shock proteins (HSP60, HSP70, and HSP90), antigen presentation proteins (MHC I and MHC II), and integrins [7, 100]. Moreover, some disease-related proteins, including Ep-CAM, epidermal growth factor receptor (EGFR), survivin, and IGF-1R, which are distributed on the surface of exosomes, can be used as biomarkers in clinical diagnosis and prognosis [101]. It is appealing that these proteins can be used both as biomarkers in biofluids and for the isolation and purification techniques introduced above. The lipid component of exosomes differs from that of the plasma membrane of the parent cells, partly because exosomes also carry Golgi-derived lipids. For example, glycosphingolipids, cholesterol, phosphatidylserine, and ceramide are abundant in exosome membranes [84, 102]. The lipid composition determines the unique rigidity of exosomes.

### Exosome heterogeneity

Exosome heterogeneity is generally characterized by differences in size, content, functional impact on recipient cells, and cellular origin. Recently, an emerging theory has classified EVs into two main types, i.e., ectosomes, which have diameters ranging from 50 to 1000 nm, and exosomes, which range in size from 40 to 160 nm [103]. Thus, challenges to effective and thorough exosome isolation once again emerge owing to the size overlap between ectosomes and endosomes. Size heterogeneity can be regulated by uneven invagination of the bounding membrane of MVBs, resulting in different amounts of fluid and solid components within exosomes [72, 104, 105]. The inherent biology of the cells and their microenvironment may regulate the repertoire of exosomal biological markers and exosome contents. The material encapsulated by exosomes contains various types of cargo, which is directly linked to exosomal heterogeneity. It has been proven that exosomes carry membrane proteins, cytosolic and nuclear proteins, extracellular matrix (ECM) proteins, nucleic acids (including mRNA, ncRNA, and DNA), and metabolites [106, 107]. Proteomic analyses of EVs have revealed heterogeneity in exosomal markers, highlighting the usefulness of this heterogeneity in experimental studies based on marker-dependent purification methods [25]. The source of exosomes can also influence their heterogeneity, and exosomes derived from different tissue types or organs possess different biological activities, and this feature also applies to cancer cell-derived exosomes [108]. It has been demonstrated that exosomal production by cancer tissue is much higher compared with that of non-cancer tissue close to the carcinoma. Although the majority of exosomes secreted by cells share a similar size, composition, and even content, exosomes derived from different cells can exert completely different effects. For example, the effects of exosomes on recipient cells can vary due to differences in the expressed cell surface receptors, which further contributes to the functional heterogeneity of exosomes. The same exosomes can induce different cellular responses in different target cell types, including promoting cell survival or apoptosis or exerting immunomodulatory functions. The combination of all of these types of heterogeneity imparts exosomes with higher-order complexity.

Investigation of the processes underlying exosomal biogenesis will help to clarify the mechanisms of tumor progression, potentially providing insight to improve cancer treatment. Variation in exosomal composition makes exosomes useful as specific probes for the diagnosis and prognosis of a variety of cancer types. Furthermore, personalized treatment will be more widely used as it is bolstered by accumulating knowledge of exosomal heterogeneity.

### Exosome-mediated intercellular communication in cancer

Exosomes are emerging as critical messengers in the intricate intercellular communication involved in cancer progression as they can transfer information among tumor cells or to other malignant or normal cells. Recent approaches based on real-time exosome tracking systems suggest that exosomes may serve as effective vehicle-mediated transfer factors both in vitro and in vivo [109, 110]. In addition, in vivo imaging has revealed that exosomes released by malignant tumor cells are taken up by less malignant cells in the same tumor and in distant tumors [10].

Mounting evidence has proven that specific cellular components derived from the original tumor cells accumulate in exosomes and that exosomes can then mediate functional responses via interactions with target tumor cells and by re-programming various types of cancer cells [13]. For example, exosomes isolated from mutant KRAS-expressing colon cancer cells enhanced the invasiveness of KRAS wild-type recipient cells. KRAS-mutant cells exert dramatic non-cell-autonomous effects on neighboring and distant cells via exosome release [111, 112]. Breast cancer cell-derived exosomes containing several precursor miRNAs along with Dicer, AGO2, and TRBP, have been found to efficiently mediate rapid silencing of mRNAs to reprogram the target cell transcriptome, thus leading to genotypic and phenotypic changes in the non-malignant target cells [91]. Exosomes can also mediate dynamic feedback between tumor cells and surrounding cells in the tumor microenvironment. It also has been elucidated that cancer-derived exosomes can modulate the phenotypic state of the surrounding cells to support tumor progression. In melanoma, tumor-derived exosomes can permanently educate bone marrow progenitor cells toward a pro-vasculogenic and pro-metastatic phenotype via the MET receptor. Transfer of the MET oncoprotein from tumor-derived exosomes to bone marrow progenitor cells promotes metastasis [113]. In female esophageal carcinoma, exosomal FMR1-AS1 secreted from esophageal carcinoma cancer stem cells (CSCs) can transfer stemness phenotypes to recipient non-CSCs in the tumor microenvironment, thereby supporting the maintenance of a cancer stem-like cell dynamic equilibrium via TLR7/NFκB/c-Myc signaling [114]. Another study reported that triple-negative breast cancer (TNBC) cells can activate stromal cells by releasing exosomes containing unshielded RNAs that mimic viral components to co-opt anti-viral immune responses, thereby promoting tumor growth [115]. Similarly, hepatocellular carcinoma (HCC)-derived exosomes can mobilize normal hepatocytes and promote motility of immortalized hepatocytes via transfer of oncogenic proteins and RNAs [116]. Furthermore, accumulating studies have reported that stromal cells in the microenvironment impart rapid expansion information to recipient cells via exosome transport. In pancreatic cancer, exosomal miR-5703 derived from pancreatic stellate cells has been linked to pancreatic tumor progression via activation of the PI3K/Akt pathway [117]. Cancer-associated fibroblasts (CAFs) are a prominent component of tumor microenvironments, and they can regulate tumor progression by transferring exosomes to neighboring cells. For example, miR-34a-5p in CAF-derived exosomes contributes to cancer proliferation and metastasis in oral squamous cell carcinoma (OSCC) [118]. In colorectal cancer (CRC), lncRNA H19 is delivered by exosomes secreted from CAFs in the tumor microenvironment, thereby influencing the stemness and chemoresistance of CRC [119]. Tumor-associated macrophages (TAMs) are a major component of tumor microenvironments. It has been reported that TAM-derived exosomes mediate intercellular transfer of ApoE, which then activates the PI3K-Akt signaling pathway in the recipient cancer cells to promote gastric cancer migration [120]. In HCC, exosome-mediated transfer of functional CD11b/CD18 protein from TAMs to tumor cells might boost their migratory potential [121]. Furthermore, CAF-derived exosomes contain intact metabolites, including amino acids, lipids, and TCA-cycle intermediates, which are internalized by prostate cancer cells to promote tumor growth [122]. More interestingly, exosomes have been shown to potentiate their own uptake. For example, melanoma-derived exosomes facilitate their own uptake by blocking cholesterol 25-hydroxylase (CH25H), an oxysterol, in defense against education of normal cells by tumor-derived exosomes [123].

To summarize, the efficient exchange of cellular components via exosomes can inform important functions in cancer development, and this activity might be useful for designing exosome-based therapeutics.

## Exosome functions in cancer

Local and distal cellular communication are important for both normal and tumor cells. Exosomes, as a means of intercellular communication, play important roles in several key oncogenic processes, including tumor metastasis, therapeutic resistance, and immune responses. The functions of exosomes are determined by the specific cargo that they deliver. Exosomes and their specific cargo, including proteins, metabolites, and nucleic acids, can provide information on potential regulatory drivers of tumor progression.

### Exosome-mediated cancer metastasis

Cancer cells, which can migrate to local or remote organs, depend on their invasion and metastasis capabilities. During metastatic progression, exosomes can act as messengers that influence important functions in multiple steps of the metastatic cascade, including angiogenesis, migration, epithelial-to-mesenchymal transition (EMT), and establishment of a pre-metastatic niche (PMN) [124]. A comparative proteomic analysis of exosomes found that exosomes contain different protein cargo based on the host cell’s metastatic properties. In this study, metastatic cell-derived exosomes contained proteins that promote migration, proliferation, invasion, and angiogenesis, while the non-metastatic cell-derived exosomes contained proteins involved in cell–cell/cell–matrix adhesion and polarity maintenance [125]. RNA deep sequencing and proteomic analysis revealed that exosomes derived from metastatic HCC cell lines carried a large number of protumorigenic RNAs and proteins, such as MET, S100 family members, and caveolins [116].

Tumor-derived exosomes can directly influence metastasis via the secretion of metastatic inducer molecules, e.g., TGF-β, SMAD3, or ncRNAs [9]. This promigratory effect of exosomes has been observed in various cancers, including pancreatic cancer, gastric cancer, liver cancer, and renal cell carcinoma (among others) [126–130]. Lymphatic metastasis is the most common form of metastasis in cancer. Exosomal miRNA and protein levels have also been found to be closely associated with lymphatic metastasis in cancer patients [126, 131, 132]. In addition, exosomes from tumor cells that undergo EMT can stimulate neighboring cells to acquire EMT-like features. In liver cancer, treatment of low metastatic cancer cells with exosomes isolated from highly metastatic cancer cells resulted in an EMT-like phenotype and increased migratory and invasive features accompanied by decreased expression of the epithelial marker E-cadherin [133].

Furthermore, several cell types in tumor microenvironments, e.g., macrophages and CAFs, have been shown to play key roles in cancer metastasis via exosomes. In CRC, M2 macrophage-regulated CRC cell migration and invasion depends on M2 macrophage-derived exosomes [134]. In liver cancer, macrophages might exert effects by secreting miR-92a-2-5p in exosomes to decrease liver cancer cell AR expression, which then leads to increased liver cancer cell invasion [135]. CAF-secreted exosomes play a key role in promoting breast cancer motility and metastasis by mobilizing autocrine Wnt-PCP signaling in tumor cells [136]. In addition, in CRC, CAFs promote stemness and EMT in the cancer cells by directly transferring exosomes, leading to a significant increase in the miR-92a-3p level [137].

The formation of PMNs, which involves a series of events that prepare future metastatic sites for incoming tumors and supports engraftment and survival of metastatic cells, has been shown to rely on exosomes [138]. In pancreatic ductal adenocarcinoma (PDAC), malignant exosomes play a key role in the generation of liver PMNs. Kupffer cells (KCs) in the liver can selectively uptake exosomes, subsequently promoting the formation of pro-inflammatory milieus that support metastasis [139]. A crucial initial step in PMN generation in target organ tissue involves angiogenesis. Multiple studies have demonstrated that exosomes are involved in angiogenesis and increased vascular permeability, both of which facilitate PMN formation [113, 140]. Multiple types of bone marrow-derived cells (BMDCs) promote ECM remodeling in PMNs by releasing exosomes, thereby promoting PMN formation [141, 142]. For example, primary melanoma-derived exosomal RNAs, which activate TLR3 to recruit neutrophils, promote lung PMN formation [143]. Interestingly, an exosome-based artificial PMN that impairs crosstalk between metastatic cells and their environment has been shown to disrupt metastasis and to have a statistically significant benefit on survival outcomes [144].

More interestingly, exosomes are specific to the recipient cell type and are subject to organotropic metastasis. For example, breast cancer-derived exosomes are taken up by endothelial cells in the brain and by fibroblasts in the lungs [145, 146], whereas pancreatic cancer-derived exosomes are taken up by Kupffer cells in the liver [139]. EGFR carried in exosomes secreted from gastric cancer cells can be delivered to the liver and integrated into the plasma membrane of liver stromal cells, thus favoring the development of a liver-like microenvironment and promoting liver-specific metastasis [147]. Lyden and colleagues reported that tumor exosome integrins can control organotropic metastasis by fusing with organ-specific resident cells to establish PMNs by activating Src phosphorylation and pro-inflammatory S100 expression. Exosomal integrins α6β4 and α6β1 were associated with lung metastasis, while exosomal integrin αvβ5 was linked to liver metastasis [146]. A recent paper found that CD44 variant isoform v6 (CD44v6) in exosomes released by pancreatic and CRC-initiating cells contributes to tumor progression by interacting with α6 and β4 integrins, leading to enhanced cell migration and invasion in the recipient cells [148].

Additionally, hypoxia, a crucial factor in tumor microenvironments, is beneficial to tumor metastasis. Haiou Yang et al. reported a difference between the metastatic potential of hypoxic cancer cells and that of relatively normoxic cancer cells [149]. Hypoxia promotes exosome release by breast cancer cells, and this process might be regulated by hypoxia-inducible factor 1-a (HIF1-a) [150]. During hypoxia, bladder cancer cells can release oncogenic lncRNA-UCA1-enriched exosomes into the ECM, leading to remodeling of unfavorable microenvironments to promote tumor development [151]. Under hypoxic conditions in lung cancer, exosomal miR-23a was significantly upregulated, resulting in increased vascular permeability and cancer transendothelial migration by targeting prolyl hydroxylase and tight junction protein ZO-1 [152]. Hypoxia-resistant multiple myeloma cells produce more exosomes than do the parental cells under normoxic or acute hypoxic conditions. Exosomal miR-135b released from hypoxic multiple myeloma cells promotes angiogenesis by targeting factor-inhibiting HIF-1 (FIH-1) [153].

Conversely, exosomes might also inhibit tumor metastasis. Exosomes from TWEAK-stimulated macrophages significantly inhibited metastasis of epithelial ovarian cancer [154]. In addition, exosomes released from poorly metastatic cancer cells can potently inhibit metastasis. "Non-metastatic" exosomes induce cancer cell clearance in PMNs via the recruitment of NK cells and TRAIL-dependent killing of melanoma cells by macrophages [155].

### Exosomes in tumor-associated immune regulation

**Fig. 1:**
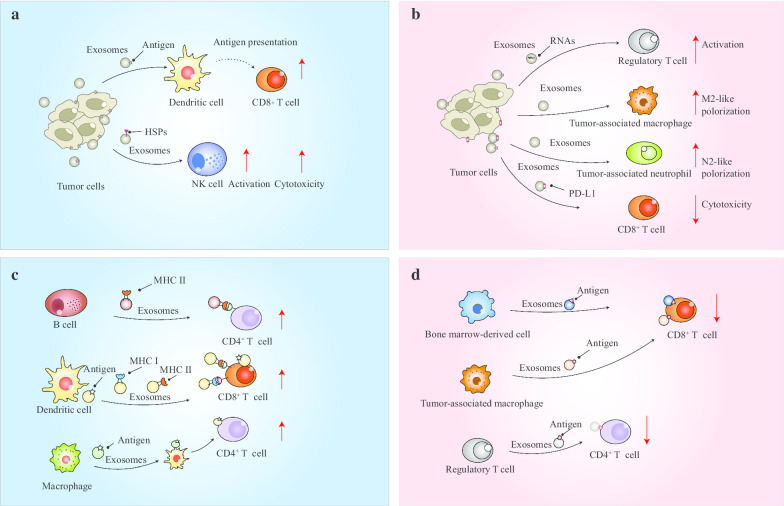
Exosomes from tumor or immune cells stimulate or suppress anti-cancer immunity. **a** Tumor-derived exosomes bearing antigens stimulate CD8^+^ T cells via dendritic cells (DCs). HSPs-containing exosomes promote the activation and enhance the cytotoxicity of NK cells. **b** RNA-containing exosomes activate regulatory T cells. Tumor-derived exosomes can stimulate tumor-associated macrophages to adopt a tumor-promoting M2-like phenotype. Similarly, tumor-derived exosomes promote N2-like polarization in tumor-associated neutrophils. PD-L1-bearing exosomes inhibit the cytotoxicity of CD8^+^ T cells. **c** B cell-derived exosomes carrying MHC II stimulate CD4^+^ T cells by specifically targeting receptors on CD4^+^ T cells. DCs present antigens and MHC I to CD8^+^ T cells, resulting in their activation. Macrophages present antigens to CD4^+^ T cells via DCs, ultimately triggering CD4^+^ T cells activation. **d** Antigen-bearing exosomes suppress the function of CD8^+^ T cells and CD4^+^ T cells in bone marrow-derived cells, tumor-associated macrophages, and regulatory T cells

How tumors evade immune recognition is a cornerstone in our understanding of cancer biology. Recently, the roles of exosomes in immune modulation during cancer progression have gained great attention. As pivotal mediators of intercellular communication and immunological function, exosomes have been shown to regulate the functions of cytotoxic T cells [156], NK cells [157, 158], TAMs [159], neutrophils [160, 161], myeloid-derived suppressor cells (MDSCs) [162], dendritic cells (DCs) and Treg cells [163] (Fig. 1). These modulatory effects mainly depend on immune-related ncRNAs, proteins, and other immune molecules expressed on exosomes, e.g., peptide-bound MHC class I and II, and T cell stimulatory molecules.

A large body of evidence has demonstrated that exosomes promote pro-tumorigenic phenotypes by facilitating immunosuppression. Immune suppression by exosomes has been shown to suppress T cell function and NK cell activity and to stimulate MDSCs. For example, PD-L1 is localized on the surface of tumor-derived exosomes in plasma samples from patients with a variety of cancers [164]. Exosomal PD-L1 inhibits T cell function and attenuates the anti-cancer immune response, thus facilitating tumor growth [165, 166]. In addition, abundant studies have demonstrated that tumor-derived exosomes can modulate the cell biology of MDSCs, including increasing their expansion, promoting their activation, and enhancing their immunosuppressive function [162]. Tumor-associated neutrophils (TANs) play pro- or anti-tumor roles depending on their phenotypes in tumor microenvironments [167]. Studies have demonstrated that tumor-derived exosomes can increase the number of tumor-infiltrating neutrophils and induce pro-tumorigenic N2-like polarization, which accelerates tumor proliferation and inhibits the immune response [160, 161]. Tumor-derived exosomes can also reduce T cell proliferation and cytotoxic functions directly and/or indirectly by inhibiting DCs. Studies have demonstrated that exosomes create immunosuppressive microenvironments by blocking DC differentiation and maturation via the IL-6-STAT3 signaling pathway and by inhibiting differentiation of myeloid precursor cells into CD11c^+^ DCs and inducing apoptosis, which promote immune suppression of DCs and decreases T cell activity [168–170]. In addition, endogenous miR-155 and miR-146a, two important miRNAs that regulate inflammation, are released from DCs in exosomes and are subsequently taken up by recipient DCs, which then mediate the inflammatory response [171]. It was demonstrated that breast cancer-derived exosomes can directly transmit lncRNA SNHG16 to induce CD73^+^γδ1 Treg cells, which are the predominant regulatory cell population in tumor microenvironments that promote tumor progression [163]. Furthermore, Treg-derived EVs, including exosomes, can regulate DC function via the induction of a tolerogenic phenotype [172]. Studies have demonstrated that BMDC-derived exosomes containing PD-L1 can inhibit CD8^+^ T cell activation and proliferation in vitro and in vivo in tumor-bearing mice [173]. In epithelial ovarian cancer, TAM-derived exosomes mediate the interaction between TAMs and T cells, generating an immune-suppressive microenvironment that facilitates ovarian cancer progression and metastasis by causing a Treg/Th17 cell imbalance [174]. Recent studies have reported that exosomes secreted by mesenchymal stem cells (MSCs) drive accelerated breast cancer progression by inducing differentiation of monocytic myeloid-derived suppressor cells into highly immunosuppressive M2-polarized macrophages [159].

Recently, exosomes derived from various cell types have been shown to play crucial roles in antigen presentation and T cell activation, thereby promoting immunity. For example, exosomes carrying tumor-specific antigen can support antigen presentation by antigen-presenting cells (APCs) and stimulate the activation of an anti-tumor immune response [175, 176]. It has been demonstrated that uptake of tumor exosomes can increase DC maturation and activation, leading to enhanced levels of CD11c and MHC class I and II. In addition, exosomes secreted by tumor cells can activate DCs and increase the number of CD8^+^ T cells by elevating the expression of the costimulatory factors CD80 and CD86 and intercellular adhesion molecules on DCs [177, 178]. As soon as they recognize tumor-specific antigens on DCs, CD8^+^ T cells are activated followed by their differentiation into effector cytotoxic T lymphocyte (CTL). Next, the CTLs infiltrate tumor lesions and attack tumor cells via specific interactions. During this process, DC-secreted and Treg cell-secreted exosomes, respectively, stimulate and inhibit CTL generation and cytotoxic activity [163, 179, 180]. Exosomes have also been shown to transfer functional MHC complexes to DCs, thereby granting them a significant antigen-presenting ability [181]. Exosomes from knock-out mice lacking the MHC class II-peptide complex resulted in significant abrogation of the suppressive effect [182]. As innate immune cells, NK cells play essential roles in rapid immunity to orthotopic and metastatic tumor cells, and efforts have been undertaken to effectively leverage their antitumor properties. It has been demonstrated that HSP70-positive exosomes secreted from tumor cells can activate the cytotoxic response of NK cells, resulting in reduced tumor growth [183]. Furthermore, NK cell-derived exosomes can also exert cytotoxic effects on tumor cells; thus, they warrant further exploration for development as a potential anti-tumor strategy [184]. In neuroblastoma, NK cell-derived exosomes carrying the tumor suppressor miR-186 are cytotoxic to MYCN-amplified neuroblastoma and inhibit tumor escape mechanisms [185]. In addition, active T cells can release bioactive exosomes that attenuate tumor invasion and metastasis [186]. In melanoma, Ag-specific CD8^+^ T cells can modulate immune responses via T cell-released bioactive exosomes through regulation of peptide/MHC class I and Fas ligand-mediated cytotoxicity [187]. In summary, tumor- and immune cell-derived exosomes can exert tumor-associated immunomodulatory effects by delivering immune-stimulatory or immune-suppressive signaling molecules, thereby regulating cancer progression.

### Exosomes and drug resistance in cancer

Although an increasing number of novel antitumor drugs and ever-improving therapeutic strategies are providing promising benefits to cancer patients, high therapeutic resistance remains a major obstacle for effective cancer treatment. Analyses of experimental models and patient tumors have demonstrated that exosomes are involved the development of therapeutic resistance in cancer [188].

Originally, it was shown that drug-resistant cells can transfer resistance to sensitive cells via exosomes both in vitro and in vivo [189, 190]. A large body of evidence currently indicates that bioactive exosomal cargo, such as proteins, ncRNAs, and mRNAs, affect drug resistance, and mechanistic insight is emerging. For example, in renal cell carcinoma, EV fractions that contain exosomes can shuttle miRNA from chemotherapy-resistant tumor cells to sensitive tumor cells, which then become resistant via acquisition of resistance information [191]. Likewise, imatinib-resistant chronic myeloid leukemia (CML) cell-derived exosomes carrying resistance information in the form of miR-365 can be internalized by sensitive CML cells, which then become resistant [192]. The lncRNA ARSR carried in exosomes shed by tumor cells can induce a phenotypic transformation from sunitinib sensitivity to resistance [193]. Furthermore, exosomal circUHRF1 enhances HCC resistance to anti-PD1 therapy via increased expression of T cell immunoglobulin and mucin domain 3 (TIM-3), a negative immunomodulatory receptor that interacts with tumor ligands [194].

Notably, exosome-mediated stromal communication with cancer cells can influence treatment responses. Under gemcitabine treatment-imposed stress, CAFs significantly increase their secretion of exosomes that can target recipient cells to promote tumor proliferation and drug resistance [195]. Paracrine exchange of exosomal miRNAs between neuroblastoma cells and neighboring human monocytes can affect chemotherapy resistance [196]. In breast cancer, stromal cells use exosomes to orchestrate intricate crosstalk between cancer cells to drive chemotherapy and radiation resistance [197]. CAFs can promote chemotherapy resistance in CRCs by increasing the miR-92a-3p level in the recipient cells via secretion of exosomes loaded with miR-92a-3p [137]. In ovarian cancer, miR-223 was found to be enriched in exosomes released from macrophages under hypoxia, and these exosomes could be transferred to epithelial ovarian cancer cells to promote ovarian cancer chemoresistance [189]. In leukemia, bone marrow stromal cell-derived exosomes carrying fibroblast growth factor 2 (FGF2) can be endocytosed by leukemia cells, endowing the leukemia cells with protection from tyrosine kinase inhibitors [198].

Recent studies have reported that exosomes can also reduce the effects of chemotherapy via removal of chemotherapeutic drugs from tumor cells. For example, breast cancer cell-derived exosomes can reduce the effectiveness of trastuzumab, a first-line drug for advanced HER2-positive breast cancer patients; thus, removal of such exosomes from circulation could restore trastuzumab sensitivity in the breast cancer cells [199]. Therefore, exosomes are a major determinant for inducing or disseminating resistance phenotypes in anti-tumor therapy.

Taken together, the findings summarized here have established that exosomes can exert functional effects on other cells or host cells to support all stages of cancer progression. A better understanding of these functions will support the development of critical exosome-informed therapies with expanded efficacy in cancer treatment.

## Clinical applications of exosomes in cancer

**Fig. 2:**
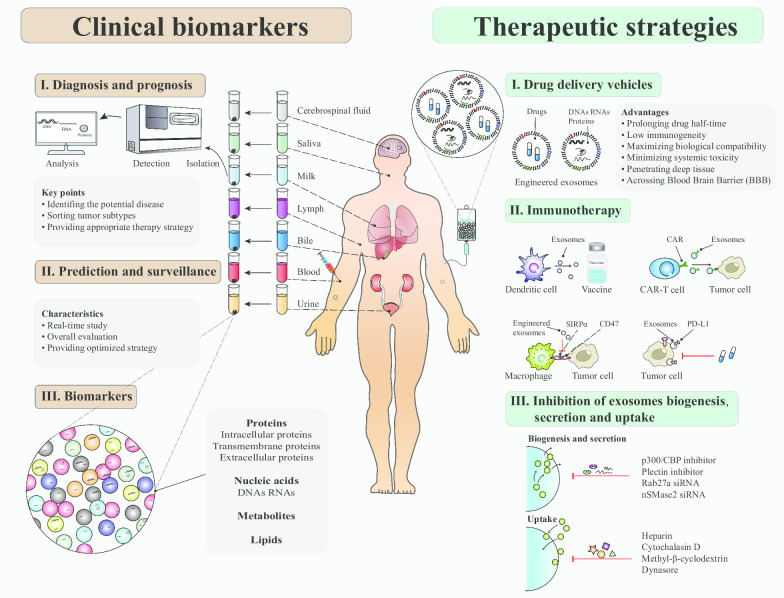
Clinical applications of exosomes in cancer. Exosomes can be extracted from bodily fluids, including cerebrospinal fluid, saliva, milk, lymph, bile, blood, and urine (among others). Analysis of the molecular contents of exosomes, including proteins, nucleic acids, metabolites, and lipids, could provide unique opportunities in the context of liquid biopsies for gaining information about the presence, molecular profile, and behavior of cancer. Exosomes can be used as biomarkers in cancer diagnosis, prediction, and surveillance. Clinical treatment mainly involves three strategies: First, cargo, including drugs, DNAs, RNAs, and proteins, can be encapsulated in exosomes and targeted to cancer sites. Second, immunotherapy can be used in cancer therapy. DC-derived exosomes inhibit tumor progression. CAR-containing exosomes, unlike CAR-T cells, suppress tumor progression via receptor binding. The interaction between SIRPα on macrophages and CD47 on tumor cells can be blocked by engineered exosomes. Therapeutics inhibit the release of PD-L1-bearing exosomes. Finally, inhibition of exosome biogenesis, secretion and uptake are relevant to cancer therapy. Exosome secretion and biogenesis can be prevented via a p300/CBP inhibitor or genetic knockout of Rab27a and nSMase2. The uptake process can be prevented by inhibitors such as heparin, cytochalasin D, methyl-β-cyclodextrin, and dynasore

The known key roles of exosomes in promoting tumor metastasis, chemoresistance, and immunity demonstrate that knowledge of exosomes is not only important for understanding the significance of cancer progression, but that it can also provide useful information to clinicians (Fig. 2).

### Exosomes as diagnostic and predictive biomarkers for cancer

Exosomes carry various types of cargo, including mutated DNA fragments, RNAs, and protein signatures that are associated with various phenotypes. The billions of exosomes circulating in bodily fluids provide a great deal of information about an individual’s tumor state. Recently, exosomes have emerged as a novel tool for the development of liquid biopsies to follow cancer progression and cancer treatment.

Based on an analysis of a large number of serum samples, the level of exosomal glypican-1 (GPC1) in the serum of pancreatic cancer patients was found to be significantly higher than that in healthy individuals, highlighting an important opportunity for the use of exosomes to detect early pancreatic cancer [200]. Further studies indicated that the level of exosomal GPC1 might be an attractive non-invasive diagnostic and screening tool in a variety of cancers [200, 201]. A multitude of evidence revealed that distinct exosomal proteins, e.g., Rab, GTPases, ESCRT, CD9, CD81, CD63, flotillin, TSG101, ceramide, Alix, tetraspanins, and integrins, could be used for cancer detection and consideration of clinical outcomes in cancer patients [7] (Table 2). Recently, via a proteomic analysis of EVs and other particles, including exosomes, from 426 human samples, David Lyden’s team found that pan-EVs and particles can carry cargo that can be used to classify ambiguous primary tumor types and which might serve as reliable biomarkers for cancer detection and determining cancer type [202].

**Table 2:** Proteins on exosomes as biomarkers in cancer

Cancer	Proteins	Samples	Biological effects	Refs.
Pancreatic cancer	Glypican-1 CKAP4 Eps8 ZIP4	Serum Serum Serum Serum	Unknown Promoting cells proliferation and migration Promoting tumor metastasis Promoting tumor growth	[200] [213] [214] [215]
Colorectal cancer	CPNE3 TMEM180	Plasma Supernatant	Unknown Uptaking or metabolizing glutamine and arginine	[216] [217]
Breast cancer	AnxA2 CD82 HSP70 MTA1 TRPC5	Serum Serum/Plasma Blood Serum Blood	Promoting angiogenesis Inhibiting tumor cells metastasis Promoting tumor progression Promoting tumor progression Promoting tumor chemoresistance	[218] [219] [220] [221] [222]
Glioblastoma	PTRF	Serum	Altering tumor microenvironment	[223]
Gastric cancer	GKN1	Serum	Maintaining mucosal homeostasis and regulating cell proliferation and differentiation	[224]
	PSMA3	Serum	Promoting tumor metastasis	[225]
	PSMA6	Serum	Promoting tumor metastasis	[225]
	TRIM3	Serum	Inhibiting tumor growth and metastasis	[226]
Prostate cancer	EphrinA2 αvβ3	Serum Blood	Regulating tumor invasiveness and tumorigenesis Promoting tumor cell migration	[227] [228]
Lung cancer	ADAM10	Blood	Mediating tumor progression	[229]

In addition, serum- or plasma-derived exosomes can contain DNA useful for the identification of genetic mutations and deletions, thus providing information about cancer-specific mutations. In vivo experiments have shown that circulating exosomal DNA isolated from plasma can be used to identify mutations in parental tumor cells [97]. The EGFR^T790M^ mutation is a critical biomarker in non-small cell lung cancer (NSCLC). Detection of the T790M mutation in exosomal nucleic acid (exoNA) in plasma has been shown to be superior to detection using circulating tumor DNA (ctDNA) or circulating free DNA (cfDNA), particularly in patients with intrathoracic M0/M1a disease [203, 204]. In addition to the increased sensitivity exoNA affords for mutation detection, it has also been shown that mutations in exoNA can serve as biomarkers of clinical outcomes in cancer patients. In patients with advanced NSCLCs, low exoNA mutation allelic frequency correlates to better prognosis and is an independent prognostic factor for longer survival [203].

Since exosomal miR-21 was first discovered as a serum biomarker for cancer diagnosis and prognosis, it has become clear that exosomes harboring ncRNAs might also inform diagnosis and be useful for monitoring cancer progression [205, 206] (Table 3). For example, serum exosomal miR-301a, which is thought to be a candidate oncogene, serves as a novel diagnostic and prognostic biomarker for glioma [207]. MiR-451a, which is carried in plasma exosomes, serves as a novel biomarker for the early prediction of recurrence and prognosis in NSCLC patients after curative resection [208]. Circulating exosomal ncRNA, i.e., miRNA-21 and lncRNA ATB, are novel prognostic markers for HCC [209]. Furthermore, miRNA profiling in urine might be useful for detecting bladder cancer [210].

**Table 3:** Non-coding RNAs in exosomes as biomarkers in cancer

Cancer	Non-coding RNAs	Samples	Mechanism	Refs.
Esophageal cancer	miR-21 SeG-NchiRNA	Serum/Plasma Saliva	Targeting programmed cell death 4 and activating c-Jun N-terminal kinase Unknown	[230] [231]
Hepatocellular cancer	miR-92b miR92a-3p circPTGR1	Serum Serum Serum	Downregulating CD69 and NK cell-mediated cytotoxicity Inhibiting PTEN/Akt pathway Regulated by miR449a-MET pathway	[232] [233] [234]
Pancreatic cancer	miR-21 miR-451a miR-4525	Serum/Plasma Serum/Plasma Serum/Plasma	Unknown Unknown Unknown	[235] [236] [235]
Colorectal cancer	miR-25-3p miR-106b-3p	Blood Serum	Targeting KLF2 and KLF4 Downregulating DLC-1	[237] [238]
Breast cancer	miR-21 miR‐122‐5p miR‐215‐5p let‐7b‐5p	Urine Plasma Plasma Plasma	Unknown Downregulating syndecan-1 Regulated by Pax-5 Decreasing DNA repair capacity	[239] [240] [240] [240]
Glioblastoma	HOTAIR miR-221 miR-301	Serum Serum Serum	Unknown Targeting DNM3 Activating AKT and FAK signals	[241] [242] [207]
Gastric cancer	HOTTIP circ-RanGAP1 lncUEGC1	Serum Plasma Serum	Promoting gene transcription of several 5′ HOXA genes Mediating miR-877-3p/VEGFA Unknown	[243] [244] [245]
Prostate cancer	circ_0044516 miR-501-3p miR-1246 miR-196a-5p	Blood Urine Serum Urine	Unknown Unknown Mediating EMT Unknown	[246] [247] [248] [248]
Lung cancer	circSATB2 lncGAS5 miR-21 miR-106b	Serum Serum Serum Serum	Regulating fascin homolog 1 and actin-bundling protein 1 expression Unknown Mediating PI3K/Akt/mTOR pathway Targeting PTEN	[249] [250] [251] [252]
Bladder cancer	MALAT1 PCAT-1 SPRY4-IT1 lncUCA1 circPRMT5	Urine Urine Urine Serum Urine/serum	Unknown Unknown Unknown Mediating EMT Mediating EMT	[253] [253] [253] [151] [254]

* EMT* Epithelial-mesenchymal transition, *mTOR* Mechanistic target of rapamycin, *PTEN* Phosphatase and tensin homolog deleted on chromosome ten, *VEGFA* Vascular endothelial growth factor A

Interestingly, exosomes with the potential to be used for monitoring patient treatment responses or for early prediction of treatment outcomes have also been discovered, which could be used to support changes to treatment regimens. For example, the miR-146a-5p level in serum exosomes predicts the efficacy of cisplatin for NSCLC patients and can be used for real-time monitoring of drug resistance [211]. In patients who responded to treatment, the level of exosomal PD-L1 in the blood before treatment was significantly lower than that of the patients who did not respond to treatment, indicating that exosomal PD-L1 is associated with an anti-PD-1 response and that it might serve as a predictor for anti-PD-1 therapy [166].

Exosomal biomarkers in biofluids provide important molecular information about tumors. Unlike ctDNA and cfDNA, which have been isolated for detection despite their low concentration, exosomes are robustly and systemically distributed, supporting improved sampling and isolation [212]. While exosomes have already been used as a tool for optimizing detection methods and improving accuracy, it is clear that there are many uncharacterized biomarkers on or in exosomes that will serve as precise biomarkers for cancer detection, prediction, and surveillance as well as for the development of novel tumor therapeutics.

### Exosomes and therapeutic strategies in cancer

Once exosomes enter the recipient cell, their cargo is released. Components in the cargo can then drive changes in a variety of biological processes, including gene expression, immune responses, and signal transduction. To fight cancer cells, exosomes can be loaded with therapeutic drugs, antibodies, or RNAi designed to manipulate gene expression, which is now acknowledged as a promising approach for more efficient cancer treatment.

#### Exosomes as drug delivery vehicles

As an endogenous, membrane-permeable cargo carrier, exosomes can transfer active macromolecules, including nucleic acids and proteins, into recipient cells for cell-to-cell information exchange. Therefore, exosomes have come into focus as "natural nanoparticles" for use as drug delivery vehicles.

Recently, a large repertoire of delivery tools has been exploited, including liposomes, dendrimers, polymers, and exosomes in particular [255, 256]. However, most nanocarriers manipulated via nanotechnology for targeted therapy encounter difficulty passing the BBB, penetrating deep tissue, and in uptake by recipient cells, stemming from biological, morphological, and compositional heterogeneity [257]. Notably, exosomes are considered an ideal delivery carrier due to their ability to minimize cytotoxicity and maximize the bioavailability of drugs for a variety of diseases, including cancer. Furthermore, exosomes have many advantages as drug delivery vehicles since they are structurally stable and can maintain their stability and activity during long-term storage. The chemotherapeutic doxorubicin (Dox) loaded in breast cancer-derived exosomes is more stable and accumulates more robustly in tumors; furthermore, it is safer and more efficient than free Dox for the treatment of breast cancer and in ovarian cancer mouse models [258]. In PDAC, studies revealed that the half-life of exosomes in circulation is longer than that of liposomes [259]. Furthermore, unlike non-host vehicles, exosomes are relatively non-immunogenic; thus, they do not induce immune rejection or other complications. Furthermore, they possess an intrinsic ability to easily cross biological barriers, especially the BBB. For example, exosomes isolated from brain endothelial cells were more likely to display brain-specific biomarkers for delivery of anticancer drugs across the BBB, and their use resulted in decreased tumor growth [260].

Because the exosomal structure is characterized by a lipid biolayer and an inner aqueous space, both hydrophilic and hydrophobic drugs can be encapsulated into exosomes. The therapeutic effects of exosomes loaded with different chemotherapeutics have been shown to be more robust; for example, the beneficial effects of Dox-loaded exosomes were shown to be greater than those of Dox-loaded liposomes for reducing tumor growth in mice without the adverse effects normally associated with Dox treatment [261, 262]. Studies found that a combination of macrophage-derived exosomes and paclitaxel (PTX) had high anticancer efficacy in the pulmonary metastasis mouse model. An optimized formulation that modified PTX-loaded exosomes with aminoethylanisamide-polyethylene glycol (AA-PEG) showed much higher therapeutic outcomes compared with those of PTX dissolved in cremophor oil [263].

Exosomes are considered a reasonable vehicle to deliver miRNAs or small interfering RNAs (siRNAs) to recipient cells to help regulate the expression levels of relevant genes, particularly oncogenes, which are considered potential targets in tumor therapy. Since the first description of loading exosomes with siRNA to control gene expression in the mouse brain, many cancer-focused studies assessing the possibility of using engineered, RNA-loaded exosomes to suppress gene expression in recipient cells have followed [264]. Exosome-based RNAi therapy has higher robustness, compatibility, and stability [255]. Accumulating studies have established that delivery of miRNA or siRNA payloads via exosomes is a potential clinical tool in exosome-based therapies for the treatment of pancreatic cancer [259], breast cancer [265], among others. In PDAC, engineered exosomes carrying a specific siRNA that targets oncogenic Kras^G12D^, a commonly mutated gene, were proven be effective at suppressing tumorigenesis in multiple pancreatic cancer mouse models [259]. Currently, engineered mesenchymal stromal cell-derived exosomes carrying KRAS^G12D^ siRNA are under investigation in phase I clinical trials for the treatment of patients with metastatic pancreas cancer with the Kras^G12D^ mutation (NCT03608631).

Because they carry cell-type-specific proteins found in the membrane of their parent cells, exosomes can be modified with specific factors to target them to tumor tissue or tumor microenvironments. For example, exosomes from immature DCs modified with targeting ligands with the αv integrin-specific iRGD peptide (which acts as a recognition sequence for integrins) can be used therapeutically for the delivery of Dox to tumors; thus, this approach has high potential value for targeted tumor therapy [262]. Enveloped protein nanocages (EPNs), a novel biomimetic material that can be encapsulated in EVs (including exosomes), govern their own biogenesis and release. EPNs can package macromolecules and deliver them to target cells, highlighting their potential as an enhanced delivery platform for use in clinical application [266].

Recently, many fluorescent probes for labeling exosomes in living cells have effectively paved the way for real-time studies in exosome research, and they can be used, in particular, for monitoring dynamic changes in targeted drugs carried by exosomes in recipient cells [267].

#### Exosome-based cancer immunotherapy

Tumors evade immune surveillance by using a variety of different mechanisms to avoid detection by the immune system. Due to their immunomodulatory potential, exosomes may also be deployed in innovative immunological approaches to enhance antitumor immune responses [268].

In 1998, Zitvogel et al. first demonstrated that DC-derived exosomes expressing MHC class I and class II as well as T cell costimulatory molecules can facilitate immune cell-dependent tumor rejection [29]. Since then, much preclinical and clinical research has demonstrated that the use of DC-derived exosomes is a promising strategy for DC-based immunotherapy [269]. For example, DC-derived exosomes can trigger potent antigen-specific antitumor immune responses and reshape the tumor microenvironment in HCC mice, thus opening a new avenue for HCC immunotherapy [270]. In mouse tumor models, DC-derived exosomes maintain the essential immunostimulatory characteristics of DCs, such as sharing the ability to present antigens to T cells and inducing a more robust antitumor immune response [29]. Phase I clinical trials using autologous TAA-loaded DC-derived exosomes completed in cancer patients have highlighted the feasibility of large-scale DC-derived exosome production and safety for DC-derived exosome administration to patients [271, 272]. A second-generation autologous DC-derived exosome with highly immunogenic properties was developed for potential peptide-dependent activation of CD8^+^ T cells [273]. In a phase II trial with advanced NSCLC patients, IFN-γ-DC-derived exosomes carrying MHC class II molecules induced enhanced NK cell function and prolonged progression-free survival (PFS) [274]. In a preclinical study, human melanoma-derived exosomes containing and transferring heat shock 70 kDa protein 1A (HSPA1A) and full-length tumor antigens to DCs induced CD8^+^ T cell cross-priming and tumor rejection [30]. Aex-accumulated tumor-derived exosomes were found to contain the melanoma-associated antigen recognized by T cells (Mart1) tumor antigen, and these exosomes were used to deliver Mart1 tumor antigen to DCs derived from monocytes, highlighting Aex exosomes as a new, natural source of tumor-rejection antigens [275]. In phase I clinical trials, patients with advanced CRC were treated with Aex alone or Aex plus granulocyte–macrophage colony-stimulating factor (GM-CSF). Both therapies were safe and well tolerated; however, only Aex plus GM-CSF, and not Aex alone, induced a tumor-specific antitumor CTL response [276].

In light of the crucial crosstalk between immune cells and tumor cells, mounting studies have proposed a promising therapeutic strategy involving alteration of the tumor state via exosomes engineering to achieve therapeutic goals [277]. Previous studies reported that exosomes avoid clearance by the human immune system because they carry CD47 in their membranes. CD47, a “don’t eat me” signal activated via an interaction with signal regulatory protein α (SIRPα) on innate immune cells such as macrophages and DCs, is regarded as an innate immune checkpoint in cancer [278]. Engineered exosomes that antagonize the interaction between CD47 and SIRPα promote intensive T cell infiltration in syngeneic mouse models of cancer [279], indicating that exosome-mediated immunotherapy targeting the CD47/SIRPα axis is one of the most promising new strategies for immuno-oncology. Immune checkpoint therapies, particularly PD-1 and PD-L1 antibodies, have gained significant attention for the clinically promising benefits they offer cancer patients [280]. It has been reported that exosomal tumor-derived PD-L1 is a major regulator of tumor progression via its ability to suppress T cell activation [281, 282]. Suppression of exosomal PD-L1 inhibits tumor growth, even in models resistant to anti-PD-L1 antibodies, by inducing systemic antitumor immunity and memory [165].

Chimeric antigen receptor (CAR)-based cancer immunotherapy is a particularly promising therapeutic approach. In clinical applications involving solid tumors, CAR-modified T cell (CAR-T) therapy has seen limited success compared with its success with hematological malignancies (e.g., acute lymphoid leukemia) because of adverse events, such as cytokine release syndrome (CRS), cytokine storm, and on-target/off-tumor responses [283]. Recently, it has been broadly proposed that CAR-T cell-derived exosomes may substitute for CAR-T cells to act as powerful weapons due to their higher efficiency and lower toxicity compared with CAR-T treatment [284]. Surprisingly, this treatment was not influenced by PD-L1 on the tumor cytomembrane surface because CAR exosomes don’t express PD-1 proteins [285].

Therefore, exosomes might be leveraged for immune therapy either via sequestration of therapeutic antibodies or via elimination of vaccine-induced or adoptively transferred immune effector cells.

#### Exosome elimination and settlement in cancer therapeutics

Given that exosomes play key roles in cancer progression, inhibition of exosomal release as well as biogenesis in tumor cells and/or uptake by recipient cells has proven effective in the suppression of diverse tumor types.

Exosome internalization is a complex process that occurs mainly via endocytosis [286]. Some compounds, including heparin, cytochalasin D, methyl-β-cyclodextrin, and dynasore, have been described as endocytosis inhibitors; furthermore, they have been shown to abrogate exosome uptake, thereby suppressing tumor progression in glioblastoma, prostate cancer, breast cancer, and mantle cell lymphoma [287].

Inhibition of exosome secretion or biogenesis, like uptake abrogation, also seems to be promising in tumor therapy. Inhibitors related to exosomal genes that mediate exosome release, e.g., Rab27a and Plectin, can suppress exosome secretion, leading to tumor suppression [288]. Exosomal PD-L1 secretion can be shut down using an inhibitor of p300/CBP, which is involved in this process in tumors. The expression level of CD274 (which encodes PD-L1) was also affected because p300/CBP could not be recruited to the CD274 promoter. The potential of the dual effects of this inhibition, i.e., reduced CD274 expression and blocked exosomal secretion, in immune therapy might be fully realized when they are combined with immune checkpoint blockade [289]. Neutral sphingomyelinase 2 (nSMase2) mediates the synthesis of ceramide, one of the first molecules found to be involved in exosome biogenesis [84]. A recent study reported that PD-L1 activity involves its secretion in tumor-derived exosomes. Genetic knockout of Rab27a or nSMase2, which leads to removal of exosomal PD-L1, inhibits tumor growth, even in models resistant to anti-PD-L1 antibodies [165].

Recently, it has been shown that hybrid exosomes generated via membrane fusion of exosomes and lipids can modify the uptake ability of recipient cells. The lipid and exosome composition also determines the properties of the engineered hybrid exosomes, thus facilitating cargo loading [290].

## Conclusions and perspectives

In the last decade, there has been a substantial increase in the number of studies aimed at understanding the biology and function of exosomes in disease, especially cancer [291]. These studies established that exosomes are associated with several cancer hallmarks that influence tumor metastasis, immune modulation, and resistance to therapy [7]. Discoveries in the field of exosome biology have dramatically expanded our understanding of the major steps in cancer development. As deeper research of the heterogeneity of exosomes, their cargo, and their functions emerges, we will continue to better understand the precise and accurate characteristics of exosomes.

Based on the functional uses proposed for exosomes, it is now vital to understand how exosome isolation techniques can affect their functionality and clinical usefulness. Therefore, there is a need for standardized methods for the isolation, quantification, and analysis of exosomes and for obtaining high-purity exosomes that can be used in diverse scientific and clinical applications. It is likely impossible to develop a universal method for exosome isolation with optimized efficiency for obtaining high yields of pure exosomes from both cell culture supernatant and from complex biological fluids (e.g., blood). However, it is possible to develop standard methods that solve specific types of problems.

There is an unmet clinical need for improved liquid biopsy tools for cancer detection and monitoring. Clearly, the specific bioactive molecules contained in circulating exosomes highlight the substantial promise of using exosomes for early cancer detection, prognosis, and to guide therapy. However, false positives and negatives occur in diagnosis and prognosis using exosomes as biomarkers because of the quantity and heterogeneity of exosomes. It is important to enhance the sensitivity and specificity of exosomes as biomarkers in clinical practice.

Exosomes have yielded enticing results in cancer therapy, e.g., therapeutic cancer vaccines, based on preclinical data and on validation of good manufacturing practice processes. More importantly, the quality of exosomal vaccines has been dramatically improved in recent years. Exosome-based cancer therapy has been validated in several early-phase clinical trials. Specifically, bioengineered exosomes have great promise for use in developing exciting approaches for delivering potent antitumor payloads to cancer cells. Chemical or biological modification of exosomes may enhance or broaden their therapeutic power in cancer. However, the choice of exosome donor cell, drug loading method, aspects of carrier safety, and the use of targeting peptides on the exosome surface are important issues that remain to be addressed. Furthermore, improvement of the therapeutic potential and delivery efficiency of exosomes is needed. The clinical translation of exosome-based approaches to humans has great theoretical value and clinical significance for precise cancer diagnosis and treatment.

## Data Availability

The material supporting the conclusions of this review is included within the article.

## Abbreviations

BBB: Blood brain barrier
BMDCs: Bone marrow-derived cells
CAFs: Cancer-associated fibroblasts
CAR: Chimeric antigen receptor
CAR-T: CAR-modified T cell
cfDNA: Circulating free DNA
CML: Chronic myeloid leukemia
CRC: Colorectal cancer
CTL: Cytotoxic T lymphocyte
DCs: Dendritic cells
DG: Density gradient
Dox: Doxorubicin
EVs: Extracellular vesicles
gDNA: Genomic DNA
GPC1: Glypican-1
HCC: Hepatocellular carcinoma
lncRNAs: Long non-coding RNAs
MDSCs: Myeloid-derived suppressor cells
MSCs: Mesenchymal stem cells
MVs: Microvesicles
MVBs: Multivesicular bodies
NSCLC: Non-small cell lung cancer
PTX: Paclitaxel
SEC: Size exclusion chromatography
TAMs: Tumor-associated macrophages
TANs: Tumor-associated neutrophils
TEM: Transmission electron microscopy
Treg: Regulatory T cell
UC: Ultracentrifugation
